# High Resolution MRI Reveals Detailed Layer Structures in Early Human Fetal Stages: *In Vitro* Study with Histologic Correlation

**DOI:** 10.3389/fnana.2015.00150

**Published:** 2015-11-25

**Authors:** Rongpin Wang, Guangping Dai, Emi Takahashi

**Affiliations:** ^1^Division of Newborn Medicine, Department of Medicine, Boston Children’s Hospital, Harvard Medical SchoolBoston, MA, USA; ^2^Department of Radiology, Guizhou Provincial People’s HospitalGuiyang, China; ^3^Department of Radiology, Massachusetts General Hospital, Harvard Medical SchoolBoston, MA, USA

**Keywords:** human fetal brain, cerebral wall, neurogenesis, structural mri, histology

## Abstract

An understanding of normal fetal brain development is essential in detecting the early onset of brain disorders. It is challenging to obtain high-quality images that show detailed local anatomy in the early fetal stages because the fetal brain is very small with rapidly-changing complex structures related to brain development, including neurogenesis, neuronal migration, and axonal elongation. Previous magnetic resonance imaging (MRI) studies detected three layers throughout the fetal cerebral wall that showed differences in MR contrasts at 10 gestational weeks (GW), which is one of the earliest ages studied using MRI. Contrary to the MRI studies, histological studies found more layers at this fetal age. The purpose of this work is to study the development of brain structures from an early fetal period to an early second trimester stage using *ex vivo* MRI and compare it to histology. Special attention was paid to laminar structures in the cerebral wall. T2-weighted imaging was performed on fetal brain specimens ranging from 10 GW to 18 GW on a 4.7 tesla MR scanner. We obtained standard grayscale as well as color-coded images using weighted red-green-blue scales, and compared them with the histological images. Our study confirmed laminar structure in the cerebral wall in all the fetal specimens studied. We found that MRI detected four layers within the cerebral wall as early as 10 GW during the early fetal period (10–13 GW). Early second trimester (15–18 GW) was characterized by the emergence of subplate structures and five layers within the cerebral wall. The color-coded images were more useful than the standard grayscale images in detecting the laminar structures. Scans with appropriate parameters from a high tesla MR scanner showed detailed laminar structures even through a very small and thin cerebral wall at 10 GW *ex vivo*. A combination of high-resolution structural imaging and color-coding processing with histological analysis may be a potential tool for studying detailed structures of typical developing fetal brains, as well as fetal brains with developmental disorders as references for clinical MRI.

## Introduction

Fetal brain structure is complex and characterized by rapid growth and dynamic changes from the early stage to term. After embryo stage (which occurs before 10 gestational weeks, GW), early fetal period is considered to be between 10–13 GW, while midfetal period, otherwise known as early second trimester, occurs between 15–18 GW (Vasung et al., 2011). Better understanding of human fetal brain development is critical to assess developmental abnormalities that often cause various neurological and psychiatric disorders later in life (du Plessis and Volpe, 2002; Glenn and Barkovich, 2006a, b; Rakic, 2006; Saleem, 2013). Unlike the internationally accepted Carnegie system staging that is used to describe the human embryonic brain (https://embryology.med.unsw.edu.au/embryology/index.php/Carnegie_Stages), there is no standard staging system in place yet for the fetal period. Regardless of this fact, assessments have still been done by both histological and MRI-related studies on human fetal periods (e.g., Kostović and Vasung, 2009).

Magnetic resonance imaging (MRI) has potential to show fetal brain structures in a three-dimensional manner. In clinical practice, fetal brain MRI *in vivo* can be obtained routinely from 18 GW to term, and relies primarily on T2-weighted and diffusion-weighted sequences (Prayer et al., 2006; Brugger, 2011). However, fetal brain MRI from early fetal period to early second trimester is still dependent on* ex vivo* studies due to small brain-size, poor tissue differentiation, and frequent fetal generic movements. Postmortem MRI or MR-autopsy has proven to be a potential diagnostic alternative to conventional autopsy (Huisman, 2004; Thayyil et al., 2013) because of the advantages in imaging that allows the use of high-field magnets, smaller field of view along with high spatial resolution with increased acquisition time (Zhan et al., 2013). Even with this alternative, anatomical studies of human brain development during the early period are surprisingly scarce.

Previous *ex vivo* MRI studies focused on fetal brain structure using 0.5, 1.5, and 7.0 tesla MRI scanners (Brisse et al., 1997; Bendersky et al., 2006; Huang et al., 2009; Zhan et al., 2013). The earliest gestational weeks in these studies started at 13 GW. To our knowledge, there are only three review articles (Rados et al., 2006; Kostović and Vasung, 2009; Vasung et al., 2010) that refer to using a T1-weighted sequence on early fetal brain structure at 12 GW but without detailed information about the parameters used or the magnetic field intensity. There is only one original *ex vivo* MRI study that looked at 12 GW but it only focused on the periventricular pathway (Vasung et al., 2010). These pioneer MRI studies demonstrated that the cerebral wall has a trilaminar structure at early fetal stage (Rados et al., 2006; Kostović and Vasung, 2009) and a four to five layer structure at midfetal period (Rados et al., 2006; Kostović and Vasung, 2009; Zhan et al., 2013). On the other hand, histological studies revealed more layers in the cerebral wall (Kostović and Rakic, 1990; Kostović and Judas, 2002; Kostović and Judas, 2010; Bayer and Altman, 2006; Rados et al., 2006; Huang et al., 2009, 2013; Kostović and Vasung, 2009).

Due to rapid improvements of MRI techniques, new sequences and post-processing techniques emerged one after another. Presently, it remains unclear whether a high magnetic field or post-processing techniques can show more detailed structures. That along with a lack of information about T2-weighted sequence in early fetal brain leads to the purpose of this study which focuses on the development of fetal brain structures from early fetal period to early second trimester using *in vitro* T2-weighted sequence studies with histological comparisons. Special attention was paid to the structure of the cerebral wall.

## Materials and Methods

### Specimens

The Institutional Review Board at Boston University (BU) concluded that this research involved de-identified fetal tissues, which is not considered human subjects research, and therefore deemed this an exempt project. The Department of Anatomy and Neurobiology at BU approved the use of the specimens at the Boston Children’s Hospital (BCH) and related locations necessary for the current research to be completed.

Six human fetal brain specimens, 10–18 GW, were obtained from a brain specimen collection in the Department of Anatomy and Neurobiology, Boston University School of Medicine. Gestational ages were further confirmed based on foot length (Hern, 1984). Brain specimens came from either miscarriages or abortions. The inclusion criterion was the absence of known and/or suspected malformations. The specimens were fixed in a 4% paraformaldehyde solution, and stored in the same solution. We first imaged specimens without gadolinium (Gd-DTPA) MRI contrast agent to obtain a closer condition to *in vivo* imaging conditions. The specimens between 15 and 18 GW specimens were successfully imaged without Gd-DTPA, while the imaging contrast in the 10 and 12 GW specimens were too weak. Therefore we fixed the 10 and 12 GW specimens in another 4% paraformaldehyde solution that contained 1 mM Gd-DTPA to reduce the T1 relaxation time while ensuring that enough T2-weighted signals remained (Takahashi et al., 2013).

There was no record about the duration of fixation and postmortem times in Boston University. Unlike animal specimens that undergo perfusion processes, human specimens tend to have varied fixation and postmortem durations. It has been reported that fixation and postmortem durations affected tissue properties to some extent (Pfefferbaum et al., 2004; Dawe et al., 2009). However, conventional structural imaging was possibly less affected by fixatives compared to diffusion imaging (e.g., Pfefferbaum et al., 2004). Even though fixation processes reduce the difference of gray/white matter water mobility, the location of the gray/white matter border tends to remain in the same regions over fixation periods. As long as the location of the border remained in the same place, even with a weaker contrast, we believe it possible to detect such a border (in our case, borders of multiple layers) using our method.

### MR Imaging

Fetal brain imaging was performed on a 4.7 tesla MRI system (Bruker Biospin GmbH, Germany). Each fetal brain was immersed in Fomblin oil, and scanned with a custom-made MR coil that had an inner diameter of 60 mm. A spin-echo 2D T2-weighted (T2W) sequence was used for structural imaging. The acquisition parameters were as follows:

TR/TE = 200/8.8 ms, matrix 160 × 160 × 128, NEX = 4, 3D method, for 10 and 12 GW; TR/TE = 6360/42.5 ms, matrix 256 × 256, NEX = 16, 2D multi-slice scan, for 15 GW; TR/TE = 6000/42.7 ms, matrix 256 × 256, 2D multi-slice scan, for 17 GW. Flip angle was 90° for all specimens. Spatial resolution was set to minimum to secure enough signal-to-noise ratio (over 100): 0.13 × 0.13 × 0.13 mm for 10 GW, 0.16 × 0.16 × 0.5 mm for 15 GW, and 0.20 × 0.20 × 0.5 mm for 17–18 GW. The total acquisition time was approximately 2 h for each imaging session.

When using Gd-DTPA, we used the RARE 3D sequence with shortened TR and TE. The TR had to be much longer to achieve T2-weighted imaging contrast when Gd-DTPA was not used to shorten T1 of the sample. Thus, we used multislice 2D instead of 3D sequence for older samples. Because the T1 of the 10 GW sample was much shorter than TR (200 ms), and T2 was also shortened, we used TR/TE 200/9 ms as T2-weighted, and not T1-weighted.

Paraformaldehyde showed very high signal intensity and the Fomblin oil that was used during the scan showed no perceivable signal.

### Image Processing

**Figure 1:**
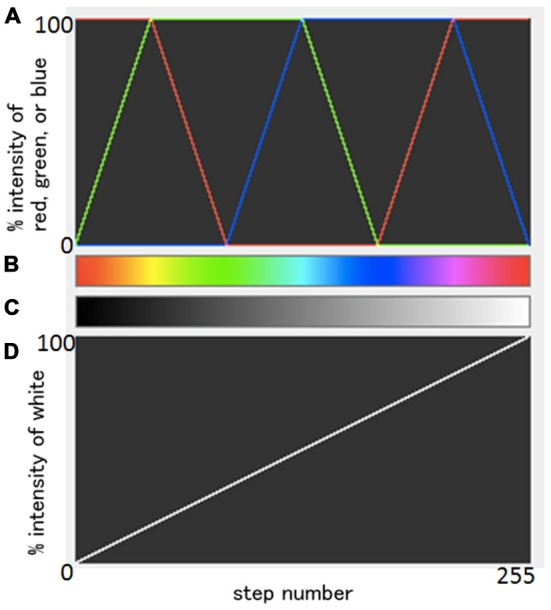
**Summary of the relationships between an original gray scale and a resulting color scale**. A scheme of the combination of three weighted original colors, red, green, and blue **(A)** and a resulting color spectrum **(B)**. Original gray scale **(C)** was generated from a simple linear change of % intensity of whiteness **(D)**. Each one of the total 256 different black/white intensities in **(C)** was transferred to a color corresponding in **(B)**.

**Figure 2:**
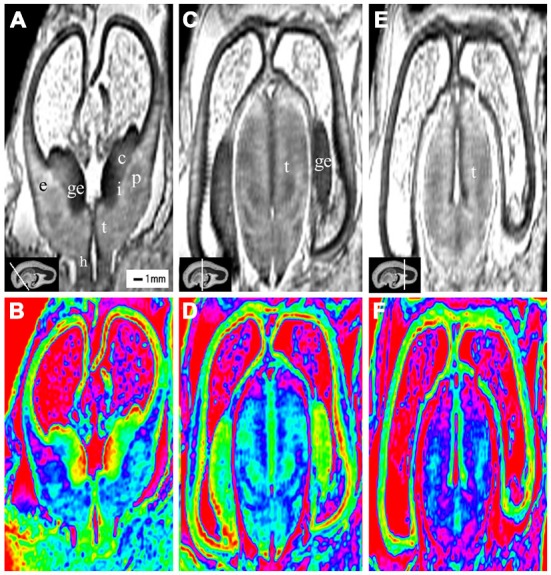
**Coronal view images of anterior, middle and posterior brain regions at 10 gestational weeks (GW)**. The lower row is the color images of corresponding parts of the upper row, which is more visible than the grayscale images in showing the laminar structure and even the nucleus structures. The anterior part consists of the anterior edge of the thalamus, basal ganglia (putamen, caudate nucleus), internal capsule, the ganglionic eminence (GE), lateral ventricles and cerebral walls whose arising part is wedge-shaped **(A,B)**. The middle part of the brain is mainly made up of the thalamus, part of the ganglionic eminence, lateral ventricles and cerebral walls **(C,D)**. The thickness of the cerebral wall in this area is heterogeneous, with the thickest part in the midlateral portion of the outer, and the thinnest in the inferior inner, which is adjacent to the ganglionic eminence **(C,D)**. The posterior part of the fetal brain mainly comprises of the thalamus, lateral ventricles and cerebral walls **(E,F)**. c, caudate nucleus; ge, ganglionic eminence; p, putamen; i, internal capsule; e, external capsule; t, thalamus; h, hypothalamus. The white line in each panel shows the location of the conresponding coronal slice.

**Figure 3:**
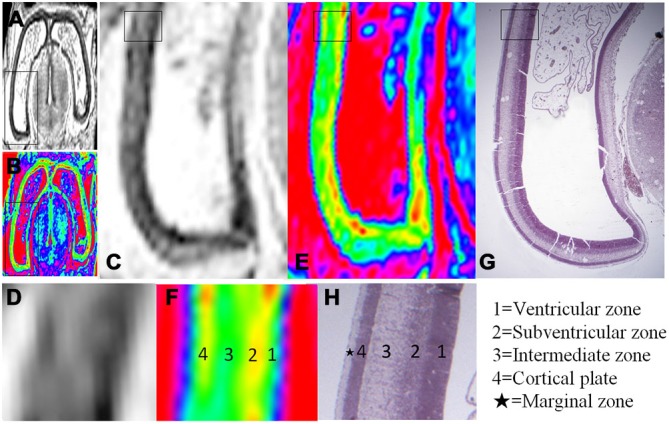
**Magnified MR images and corresponding histological images of the posterior region of the brain at 10 GW**. **(A,B)** Whole coronal sections with thick black rectangles magnified in **(C,E)**. Thin rectangles in **(C,E)** were further magnified in **(D,F)**. Thin rectangle in an hematoxylin-eosin (HE) histology image **(G)** corresponding to the region of **(C,E)** was magnified in **(H)**. All images showed four layer organization in the cerebral wall, including the ventricular zone, subventricular zone, intermediate zone and cortical plate in turn from the ventricle. The color images **(E,F)** more clearly showed the four layer organization than that of grayscale images **(C,D)**, which all correspond to histology **(G,H)** except for the marginal zone. H, histological image, H&E stained.

**Figure 4:**
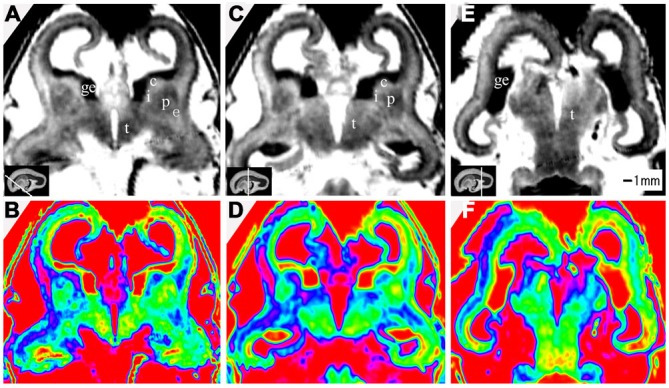
**Coronal view images of anterior, middle and posterior brain regions at 15 GW**. **(A,B)** The anterior part is composed of the anterior edge of the thalamus, basal ganglia (putamen, caudate nucleus), internal capsule, ganglionic eminence, lateral ventricles and cerebral walls whose arising part is wedge-shaped. **(C,D)** The middle part of the brain showed the thalamus, part of the ganglionic eminence, lateral ventricles and cerebral walls. The thickness of the cerebral wall is heterogeneous, with the thickest part in the midlateral portion of the outer, and the thinnest in the inferior inner, which is adjacent to ganglionic eminence. **(E,F)** The posterior regions showed the thalamus, lateral ventricles and cerebral walls. The lower row is the color color images of the corresponding parts of the grayscale images in the upper row. c, caudate nucleus; ge, ganglionic eminence; p, putamen; i, internal capsule; e, external capsule; t, thalamus. The white line in each panel shows the location of the conresponding coronal slice.

**Figure 5:**
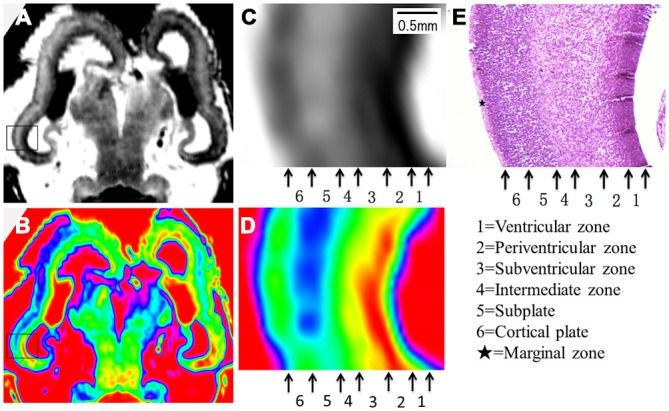
**Magnified MR images and corresponding histological images of the posterior part at 15 GW**. Rectangles in the grayscale **(A)** and color-coded **(B)** images were magnified in **(C,D)** respectively, and compared to corresponding histology image **(E)**. All images showed a six layer structure within the cerebral wall, including the ventricular zone, periventricular zone, subventricular zone, intermediate zone, subplate zone and cortical plate in turn from the ventricle, and each layer corresponds to its histological image **(E)**. E, histological image, H&E stained.

**Figure 6:**
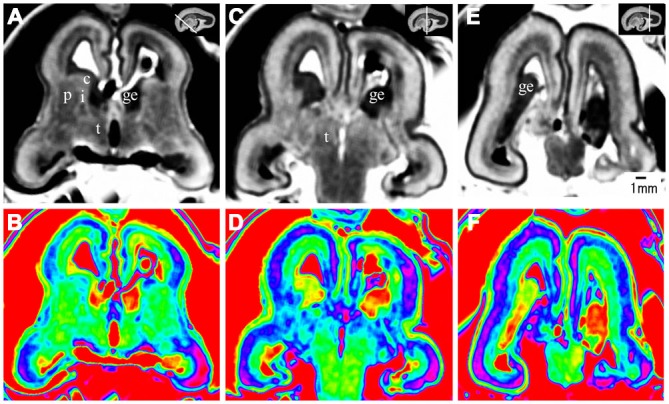
**Coronal view images going through the anterior (A,B), middle (C,D), and posterior part (E,F) of the brain at 17 GW**. The upper row, the grayscale images, clearly showed the laminar structures of the cerebral wall; the lower row is the corresponding color images of the three parts. c, caudate nucleus; ge, ganglionic eminence; p, putamen; i, internal capsule; e, external capsule; t, thalamus. The white line in each panel shows the location of the conresponding coronal slice.

**Figure 7:**
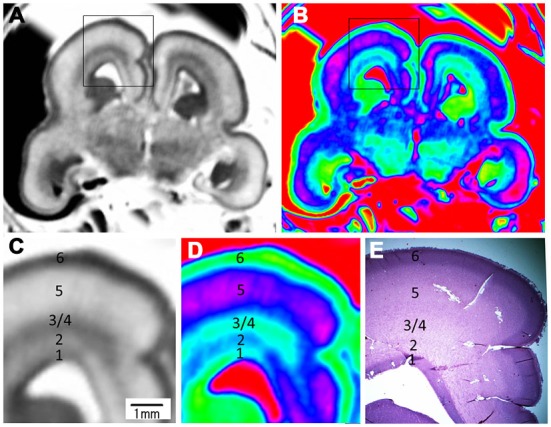
**Magnified images and corresponding histological image of the middle brain region at 17 GW**. The upper row is grayscale image **(A)** and color image **(B)**. **(C,D)** Magnified images of the rectangles in **(A,B)**. All images showed a five layer structure within the cerebral wall, including the ventricular zone, periventricular zone, subventricular zone and intermediate zone, subplate zone and cortical plate; each layer corresponds to its histological image **(E)**; the border of the subventricular zone (3) and intermediate zone (4) was not clearly differentiated even in the histological image. E, histological image, H&E stained.

**Figure 8:**
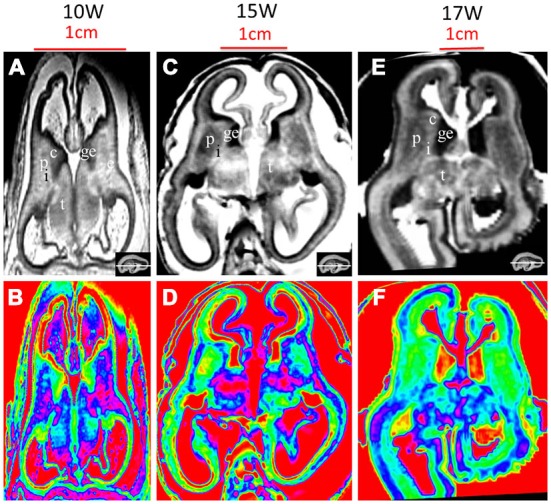
**T2-weighted axial MR grayscale images and color images at 10 GW (A,B), 15 GW (C,D) and 17 GW (E,F)**. During develoment, the cerebral walls became thicker but with heterogeneous speed; the laminar structure became clearer and the proportion of ventricles got smaller. Nucleus-rich structures such as the thalamus, putamen and caudate nucleus became enlarged and their signals were low and heterogeneous on T2-weighted grayscale images; the signal of the internal capsule and external capsule became lower, and the ganglionic eminence became enlarged. See text for details. c, caudate nucleus; ge, ganglionic eminence; p, putamen; i, internal capsule; e, external capsule; t, thalamus. The white line in each panel shows the location of the corresponding coronal slice.

Data processing was performed using ImageJ (http://rsb.info.nih.gov/ij/download.html). Standard grayscale images were first generated on a 2D viewer, and were spatially smoothed with a 3 × 3 mean filter. Next, images were loaded on a 3D volume viewer and subsequently interpolated with a trilinear function for visualization of resliced images. Then, using a transfer function tool, color-coded images were obtained with a so-called “spectrum LUT (look-up-table) style”. The transfer function from grayscale images to color-coded images is summarized in Figure 1. Combining three original weighted colors, red, green, and blue, as shown in Figure 1A; a color spectrum was generated (Figure 1B). The original gray scale (Figure 1C) was generated from a simple linear change of % intensity of whiteness (Figure 1D). Each one of the total 256 different black/white intensities in Figure 1C was transferred to a color corresponding in Figure 1B. Note that the same information was projected in both grayscale and color images, and that the chosen colormap aids visualization (i.e., human perception; Figures 2–8).

### Histology

After completion of MR experiments, the specimens were sectioned into coronal slices at 4 μm and stained by hematoxylin-eosin (HE) and Nissl at the Boston Children’s Hospital Histology Core, Department of Pathology. Then histological images were obtained under a microscope (Olympus CX41).

It should be noted that there are potential differences between the different brain regions shown in Figures 3, 3, 5 (10 and 15 weeks) and Figure 7 (17 weeks). The first two are from posterior brain regions shown in Figures 2E; Figure 4E respectively, whereas 7 is from a middle part of the brain close to the plane shown in Figure 6C. We selected the middle part of the brain in Figure 7 to match it to a good histology slice, since not all the histology slices were presentable. We noticed that the laminar structures between the different brain regions may differ to some extent. However, looking at Figures 6C,E the laminar structure of the posterior brain region shown in Figure 6E is generally similar to that of the middle brain region in Figure 6C; and such similarity was also observed in color images (Figures 6D,F).

## Results

### Cerebral Hemispheres

The most prominent structure seen during early fetal period to early second trimester was the ganglionic eminence (GE; Figures 2; 4; 8), which constituted to a part of the inner cerebral wall, protruding into the ventricles. It showed a very low signal intensity on T2-weighted images and a yellow to red color on color-coded images.

During the early fetal period (10–13 GW), many structures were recognizable as early as 10 GW. We were able to recognize different structures of the telencephalon and diencephalon from the coronal view. The anterior part consisted of the front part of the thalamus, basal ganglia (putamen, caudate nucleus), internal capsule, GE, lateral ventricles and cerebral walls (Figures 2A,B). The middle part was mainly the thalamus, part of the GE, lateral ventricles and cerebral walls (Figures 2C,D). The posterior part (behind the GE) consisted primarily of the thalamus, lateral ventricles and cerebral walls (Figures 2E,F).

The youngest specimen, at 10 GW, had four layers in the cerebral wall for both histology and MRI images (Figures 2; Figure 3). We confirmed, using histology, the layers in the following order from the ventricle to the cortex: (1) the ventricular zone, which lay close to the ventricular cavity, accounted for approximately 25% of the cerebral wall (3H), and presented as a low signal intensity on T2-weighted images and as light blue to green color on color-coded images (Figures 3C–F); (2) the subventricular zone presented as a moderate signal intensity on T2-weighted images and as a yellow band on color-coded images. It was hard to differentiate the subventricular zone from the ventricular zone on the standard grayscale images, while it was clearly delineated from the ventricular zone on the color-coded images (Figure 3); (3) the intermediate zone presented as a high signal intensity on T2-weighted images and as a green band on color-coded images (Figures 3C–F); and (4) the cortical plate, which lay near the surface of the cerebral wall and was composed of densely packed post-migratory neurons, presented as a low signal intensity on T2-weighted images and as a yellow band on color-coded images (Figures 3C–F). In addition, the thickness of the cerebral wall was heterogeneous, with the thickest section in the midlateral portion and the thinnest section in the dorsomedial portions (Figures 2; 3).

During early second trimester (15–18 GW), the cerebrum grew rapidly (Figures 4–7; Kostović et al., 2002; Kostović and Vasung, 2009). Seven layers were depicted by histology, including the ventricular zone, periventricular zone, subventricular zone, intermediate zone, subplate, cortical plate and marginal zone (Figure 5E). All layers, not including the marginal layer, were delineated on the magnified T2-weighted images and color images at 15 GW (Figures 4; Figure 5). The proportion of ventricular zone was smaller at 15 GW than at 10 GW, accounting for approximately 10% of the cerebral wall (5E).

At 17 GW, three distinctive features were found different from those at 15 GW in addition to the thickened cerebral wall. First, at 17 GW, the border of the periventricular zone was much clearer than in 15 GW, which presented relatively high signal intensity on T2-weighted images and as a blue color ribbon on color-coded images (Figures 6; Figures 7). Second, at 17 GW, the subplate zone had thickened remarkably, accounting for approximately 50% thickness of the whole cerebral wall, which presented a high signal intensity on T2-weighted images and as a pink color ribbon on color–coded images (6; Figure 7). Third, the border between the outer subventricular zone with the inner fiber layer (ingrowing callosal axons) and the ventricular zone could not be separated on MRI images (7). The increasing thickness of the cerebral wall during the early second trimester resulted in a comparatively reduced ventricular size unlike the typical laminar organization seen in early fetal brain (Figures 4–7). In addition to the inner cerebral wall thickening faster during early second trimester compared to during early fetal period, the proportion of the ventricular zone became smaller, accounting for approximately 5% of the cerebral wall, and the proportion of cortical plate got thicker at 17 GW compared to 15 GW (Figures 6; 7).

### Basal Ganglia

At 10 GW, major structures of the basal ganglia were depicted on T2-weighted MR images and color images (Figures 2; Figures 8A,B). Subcortical nuclear structures such as the thalamus, caudate nucleus and putamen all displayed relatively low signal intensity on T2-weighted images and navy blue color on color-coded images. They were separated by the developing internal and external capsule, which showed a high intensity signal on T2-weighted images and a blue color on color-coded images (2A,B). At the border of the cerebral wall and basal ganglia, the ventricular zone continued into the GE, which bulged into the ventricle and showed very low MRI signal intensity on T2-weighted images and a yellow to red color on color-coded images (Figures 2,3). On the lateral side, the GE encompassed part of the caudate nucleus which demonstrated relatively low signal intensity on T2-weighted images and a navy blue color on color-coded images (Figures 2; Figures 4; 6). As GW increased, the putamen and caudate got larger and the signal intensity got lower (2; Figure 4; 6; 8), while the range of high signal intensity of the internal and external capsules became smaller from 10–17 GW on T2-weighted images (8).

### Thalamus and Hypothalamus

The thalamus was clearly visible at 10 GW, which presented as almost homogeneous low signal intensity on T2-weighted images and as a navy blue color on color images (Figure 2). The two parts of the thalamus symmetrically surrounded the third ventricle, and the medial surface of the thalamus constituted of the upper part of the lateral wall of the third ventricle (Figures 2; Figures 4; 6; 8). With the post-conceptional age increasing from 15–17 GW, the signals of different parts of the thalamus got lower and more heterogeneous (2; Figure 4; 6; 8). The dorsal thalamus presented as a lower MRI signal intensity on T2-weighted images and as a blue color on color-coded images (8). The ventral hypothalamus showed relatively higher signal intensity on T2-weighted images and was visible as early as 10 GW (Figure 2).

## Discussion

Detailed laminar structures in the human fetal brain were successfully detected in our postmortem T2-weighted imaging. Our study demonstrated rapid growth and dynamic changes of the laminar structure from the early fetal period to the early second trimester. Developmental changes involving the number of layers and the thickness of such layers were depicted by different MR signal intensities with a color-coding scheme, which were consistent with histological sections. We detected four layers in the cerebral wall during the early period (as early as 10 GW), and five to six layers during the early second trimester, characterized by the emergence of the subplate zone. The major structures of the basal ganglia and thalamus could be delineated on T2-weighted MR images and color-coded images. The color images were visually more helpful than standard grayscale images in showing the laminar structures. To our knowledge, this is the first research study to show fetal brain structure with color-coded images.

The development of the cerebral wall is the most interesting part of the fetal brain structure. Two studies using MRI showed that the cerebral wall at early fetal period was a trilaminar structure and at midfetal period, a five-layer structure (Rados et al., 2006; Kostović and Vasung, 2009). A recent study using 7.0 tesla and T2-weighted sequences showed four layers in the midfetal period (Zhan et al., 2013). Using MRI, our study successfully imaged the cerebral wall of the early fetal period, which is characterized by a four-layer laminar organization, and the early second trimester, which is characterized by five to six layers and specialized with the presence of the periventricular zone and the subplate zone. Six layers were observed at 15 GW on both T2-weighted images and color-coded images, but five layers were found at 17 GW because the subventricular zone merged with the intermediate zone and MRI did not differentiate the border between them, which is similar to studies by Kostović et al. (2002) and Kostović and Vasung (2009). The reason that our study showed more detailed structures in the thin cerebral wall compared to Kostovićc et al. may be due to the use of high-tesla MRI with scan parameters different from theirs, as well as utilizing the use of a small custom-made MR volume coil to obtain high signal-to-noise. In contrast, a previous study using a 0.5 tesla *ex vivo* MRI found only three layers that were differentiated at 16 GW (Brisse et al., 1997). Our study also found that the development of the cerebral wall from early to midfetal period was not homogenous across brain regions, with the outer regions growing faster in thickness and volume than the inner regions. The marginal zone was not separated in our MRI images potentially because it was very thin or its signal was very close to the cortical plate but it was observed in the histologic images during our studied period.

In the developing brain, many histogenetic events (neurogenesis, gliogenesis, migration, cell differentiation, axonal elongation and synaptogenesis) proceed within laminar compartments or zones (Paus et al., 2001; Jovanov-Milosević et al., 2009). Those dynamic changes can be delineated by varying MRI signal intensities on T2-weighted sequences and different colors on color-coded images and confirmed by histology. MR signal changes associated with maturational processes can mainly be ascribed to the changes in tissue composition and organization, which occur at the histological level. MR signal changes include decreases in water content and increasing cell-density, which can be recognized as a shortening of T1- and T2-relaxation times, leading to increased T1-weighted and decreased T2-weighted intensity, respectively (Prayer et al., 2006). The ventricular zone is a germinal matrix with a high density of cells (Kostović et al., 2002; Bayer and Altman, 2006) and is compatible with our results by showing a low signal intensity on T2-weighted images (green color on color-coded images). The periventricular zone is a “waiting” position of fibers with vigorous structural plasticity due to the abundance of extracellular matrixes and guidance molecules (Judas et al., 2005), and it appeared as a relatively high signal intensity on T2-weighted images and a blue color on color-coded images (**Figure 7**; Judas et al., 2005). The intermediate zone is a migratory and axonal growth zone (Kostović and Vasung, 2009), and presented as a moderate signal intensity on T2-weighted sequence and as a pale green color on color-coded images, which encompasses both the subventricular cellular zone and the fetal white matter (Kostović and Vasung, 2009). Hence it was not differentiated from the subventricular zone at 17 GW. The subplate was characterized by a very large extracellular space and an abundant amount of different glycosaminoglycans and chondroitin sulfate proteoglycans in its extracellular matrix (Ulfig et al., 2000; Kostović et al., 2002; Kostović and Vasung, 2009). It had high proton density and therefore presented with high signal intensity on T2-weighted images and a pink color ribbon on color-coded images. The cortical plate is a cell-dense area with postmigratory neurons (Kostović and Vasung, 2009), thus it presented as a relatively low signal intensity on T2-weighted sequence and as a green color on color-coded images (Ulfig et al., 2000; Kostović and Vasung, 2009). The proportion of the ventricular zone got smaller and the signal intensity got higher on T2-weighted images going from the early fetal period to the early second trimester. Simultaneously, the cortical plate got thicker and the signal intensity got lower on T2-weighted images. These phenomena likely reflect the dynamic changes of neuronal locations as well as their migrating directions. On the other hand, the signal changes of the thalamus from almost homogeneous to heterogeneous reflected the cellular differentiation and aggregation of the nucleus during the course of development.

The dynamic change potentially related to axons was also depicted by varying MRI signal intensity on T2-weighted sequences and different colors on color-coded images. In our study, some fiber-rich tissues such as the internal capsule showed high signal intensity on T2-weighted images during early fetal period. The range of high signals of the internal capsules got narrower from 10–15 GW, and the signal intensity lowered at 17 GW, thus the color on the color-coded images changed from jade-green to navy-blue during this period (**Figure 8**). Those changes are associated with maturational processes of axons, which reflect the early dynamic change of axonal density and myelination during its development.

There are some differences between *in vivo* and *ex vivo* studies of fetal brain MRI. First, fetal brain MRI before 18 GW is mainly dependent on* ex vivo* studies due to smaller brain-size, poor tissue differentiations, and frequent fetal movements (Brugger, 2011). However, over 18 GW can be available *in vivo* or *ex vivo* studies. Second, *ex vivo* imaging provides the benefits of high resolution and high signal-to-noise-ratio at the cost of long image acquisition sessions, which, are not practical *in vivo* (Takahashi et al., 2013). High spatial resolution obtained on postmortem brains can be compared at microscopic levels due to the absence of subject movement and unlimited MRI acquisition time (Kostović and Vasung, 2009). Third, the method of specimen preparation, using a size-optimized sample container and an MR coil (Takahashi et al., 2010), and the postmortem specimens can be histologically examined and co-registered with MRI images, allowing detailed three-dimensional quantitative measurement and qualitative assessment (Kostović and Vasung, 2009). In this way, our study was able to obtain high-resolution images, which were consistent with histologic slices.

Color-coded images were transferred from grayscale intensity with a scheme shown in Figure 1; in which a band of grayscale changed into a visible color spectrum by combining the three primary colors red, green and blue. This kind of color spectrum was found to be useful for detecting laminar structures in the thin cerebral walls. For example, the ventricular zone is a cell-rich area that presented as a low intensity signal on T2-weighted images, which corresponds to the first grayscale band (Figure 1), and transferred into a green color on the color-coded images (Figure 3). The subventricular zone presented as a moderate signal intensity on T2-weighted images, which corresponds to the second grayscale band (Figure 1) and transferred into a yellow color on the color-coded images (Figure 3). It is not easy to differentiate the subventricular zone from the ventricular zone on grayscale images, but it is clearly delineated on the color-coded images (Figure 3). This is the same with the periventricular zone, which is line-shaped at 15 GW, and delineated from the ventricular zone on color-coded images (Figure 5). Thus, the color-coded images greatly enhanced our visibility to identify laminar structures. However, even with usage of spectral processing fetal laminae weren’t discernible along the entire telencephalic wall. This might be due to the spatio-temporal differences in intensity of histogenetic processes.

There were some limitations in our study. First, the sample size was very small since it had not been easy to get many whole brain specimens in early fetal stages. Second, although the thickness of each layer structure except for the most outer layer (marginal zone) was more than one voxel, it is possible that our results were affected by partial volume effects. For example, at the 10 GW, if the subventricular zone contained a whole voxel along the radial direction to the brain surface, neighboring two voxels towards the ventricular zone and towards the brain surface may have contained a part of the subventricular zone and neighboring layers, which would have caused spatial smoothing of signals from those layers. The subventricular zone could have also been across two voxels along the radial direction to the brain surface, where the two voxels likely contained both the subventricular zone and neighboring layers, which would have caused spatial smoothing of signals as above but this time both voxels contained the subventricular zone. In any case, the thicknesses of the layers detected by color-processed MR images in the current study were potentially a little thinner or thicker depending on the locations of imaging voxels across the layers. Histologically, the midfetal period brain has seven layers within the cerebral wall (Kostović et al., 2002). However, our MR images could not differentiate the border between the subventricular zone and the intermediate zone at 17 GW because the signals in the two layers were either very close to each other, or the subventricular cellular zone merged with the intermediate zone (fetal white matter) as seen in the previous study (Kostović and Vasung, 2009). It is also possible that the marginal zone was not separated in MRI images because it was very thin and the signal was similar to that of the cortical plate, or because it was affected by partial volume effect.

It should also be noted that the spectral processes used in this study did not differentiate laminar structures along the entire telencephalic wall, for example in a whole coronal plane. With the reserve of limitations already mentioned in this study, the spatio-temporal differences of MRI signal intensity might be partially driven by spatio-temporal regional variations of neuronal migration, cellular differentiation, dendritic arborization, and axonal ingrowth and outgrowth. The fact that during this period of development drastic histogenetical changes occur, it is challenging to delineate borders of transient fetal zones even on the certain histologic sections. Although this study did not explore detailed regional variations of the development of laminar structures, it is an important direction of research on human fetal brains since such variation may be a source of variety of spatially localized, specified human brain functions.

We presented T2-weighted imaging results in this study because the T2-weighted signaled significantly better-dissociated fetal brain structures than the T1-weighted signal, which is consistent with current clinical MRI (Gholipour et al., 2014). The current gold standard sequence for fetal structural imaging is a single-shot Fast Spin Echo (referred to as HASTE on Siemens scanners). Since it is a single shot sequence, its contrast is not purely T2-weighted, however, it is “T2-weighted-like” images of the fetal brain with fast imaging per slice. HASTE is rarely used in post-natal imaging, due to its sub-optimal contrast. Therefore, there is a potential that our current *ex vivo* MR findings with histology validation could be a reference for clinical fetal MRI. It is also important in future studies to test more detailed correlations between observations from MRI and histology, using multiple staining methods (e.g., see reviews by Kostović and Judas, 2002; Kostović and Judas, 2010) with multiple MR sequences.

## Conclusion

In this study, we demonstrated that a four-layer-structure could be found within the fetal cerebral wall as early as 10 GW, and five to six layers could be detected during the early second trimester using a high tesla MR scanner and appropriate parameters. The T2-weighted sequence and color-coded images proved to be as reliable as the macroscopic anatomical examination for depicting fetal brain development *ex vivo* from early fetal period to early second trimester, which enriches the appearance of MR imaging for fetal brain structures. Given that our current results might be affected by partial volume effects and was demonstrated only in limited brain regions with limited number of specimens, further studies are needed to collect more cases with different gestational ages to constitute a normal atlas at multiple different gestational weeks.

This work was supported by Boston Children’s Hospital (BCH), Guizhou Provincial People’s Hospital, and NICHD (R01HD078561, R21HD069001, R03NS091587; ET). The authors thank Donald Siwek and Darry R. Ricketts, Boston University School of Medicine for providing the fetal brain specimens from the Departmental collection, Ying Huang in the Center for Molecular Oncologic Pathology of Dana-Farber Cancer Institute for technical assistance, Borjan Gagoski at BCH for discussion, and Molly Wilkinson and Ashley Ruyan Lim at BCH for editorial assistance.

## Conflict of Interest Statement

The authors declare that the research was conducted in the absence of any commercial or financial relationships that could be construed as a potential conflict of interest.

## References

[B1] Bayer S. A. Altman J. (2006). The Human Brain During the Late First Trimester. (Boca Raton, FL: CRC Press), 352.

[B2] Bendersky M. Musolino P. L. Rugilo C. Schuster G. Sica R. E. (2006). Normal anatomy of the developing fetal brain. *Ex vivo* anatomical-magnetic resonance imaging correlation. J. Neurol. Sci. 250, 20–26. 10.1016/j.jns.2006.06.020 16905152

[B3] Brisse H. Fallet C. Sebag G. Nessmann C. Blot P. Hassan M. (1997). Supratentorial parenchyma in the developing fetal brain: *in vitro* MR study with histologic comparison. AJNR Am. J. Neuroradiol. 18, 1491–1497. 9296190 PMC8338126

[B4] Brugger P. C. (2011). “Methods of fetal MRI,” in Fetal MRI, ed. Prayer D. (New York, NY: Springer), 65–80.

[B5] Dawe R. J. Bennett D. A. Schneider J. A. Vasireddi S. K. Arfanakis K. (2009). Postmortem MRI of human brain hemispheres: T2 relaxation times during formaldehyde fixation. Magn. Reson. Med. 61, 810–818. 10.1002/mrm.21909 19189294 PMC2713761

[B6] du Plessis A. J. Volpe J. J. (2002). Perinatal brain injury in the preterm and term newborn. Curr. Opin. Neurol. 15, 151–157. 10.1097/00019052-200204000-00005 11923628

[B7] Glenn O. A. Barkovich A. J. (2006a). Magnetic resonance imaging of the fetal brain and spine: an increasingly important tool in prenatal diagnosis, part 1. AJNR Am. J. Neuroradiol. 27, 1604–1611. 16971596 PMC8139801

[B8] Glenn O. A. Barkovich A. J. (2006b). Magnetic resonance imaging of the fetal brain and spine: an increasingly important tool in prenatal diagnosis: part 2. AJNR Am. J. Neuroradiol. 27, 1807–1814. 17032846 PMC7977903

[B9] Gholipour A. Estroff J. A. Barnewolt C. E. Robertson R. L. Grant P. E. Gagoski B.. (2014). Fetal MRI: a technical update with educational aspirations. Concepts Magn. Reson. Part A Bridg. Educ. Res. 43, 237–266. 10.1002/cmr.a.21321 26225129 PMC4515352

[B10] Hern W. M. (1984). Correlation of fetal age and measurements between 10 and 26 weeks of gestation. Obstet. Gynecol. 63, 26–32. 6691014

[B12] Huang H. Jeon T. Sedmak G. Pletikos M. Vasung L. Xu X.. (2013). Coupling diffusion imaging with histological and gene expression analysis to examine the dynamics of cortical areas across the fetal period of human brain development. Cereb. Cortex 23, 2620–2631. 10.1093/cercor/bhs241 22933464 PMC3792738

[B11] Huang H. Xue R. Zhang J. Ren T. Richards L. J. Yarowsky P.. (2009). Anatomical characterization of human fetal brain development with diffusion tensor magnetic resonance imaging. J. Neurosci. 29, 4263–4273. 10.1523/JNEUROSCI.2769-08.2009 19339620 PMC2721010

[B13] Huisman T. A. (2004). Magnetic resonance imaging: an alternative to autopsy in neonatal death? Semin. Neonatol. 9, 347–353. 10.1016/j.siny.2003.09.004 15251150

[B14] Jovanov-Milosević N. Culjat M. Kostović I. (2009). Growth of the human corpus callosum: modular and laminar morphogenetic zones. Front. Neuroanat. 3:6. 10.3389/neuro.05.006.2009 19562029 PMC2697006

[B15] Judas M. Rados M. Javonov-Milosevic N. Hrabac P. Stern-Padovan R. Kostović I. (2005). Structural, immunocytochemical, and mr imaging properties of periventricular crossroads of growing cortical pathways in preterm infants. AJNR Am. J. Neuroradiol. 26, 2671–2684. 16286422 PMC7976217

[B16] Kostović I. Judas M. (2002). Correlation between the sequential ingrowth of afferents and transient patterns of cortical lamination in preterm infants. Anat. Rec. 261, 1–6. 10.1002/ar.10069 11984786

[B17] Kostović I. Judas M. (2010). The development of the subplate and thalamocortical connections in the human foetal brain. Acta Paediatr. 99, 1119–1127. 10.1111/j.1651-2227.2010.01811.x 20367617

[B18] Kostović I. Judas M. Rados M. Hrabac P. (2002). Laminar organization of the human fetal cerebrum revealed by histochemical markers and magnetic resonance imaging. Cereb. Cortex 12, 536–544. 10.1093/cercor/12.5.536 11950771

[B19] Kostović I. Rakic P. (1990). Developmental history of the transient subplate zone in the visual and somatosensory cortex of the macaque monkey and human brain. J. Comp. Neurol. 297, 441–470. 10.1002/cne.902970309 2398142

[B20] Kostović I. Vasung L. (2009). Insights from *in vitro* fetal magnetic resonance imaging of cerebral development. Semin. Perinatol. 33, 220–233. 10.1053/j.semperi.2009.04.003 19631083

[B21] Paus T. Collins D. L. Evans A. C. Leonard G. Pike B. Zijdenbos A. (2001). Maturation of white matter in the human brain: a review of magnetic resonance studies. Brain Res. Bull. 54, 255–266. 10.1016/s0361-9230(00)00434-2 11287130

[B22] Pfefferbaum A. Sullivan E. V. Adalsteinsson E. Garrick T. Harper C. (2004). Postmortem MR imaging of formalin-fixed human brain. Neuroimage 21, 1585–1595. 10.1016/j.neuroimage.2003.11.024 15050582

[B23] Prayer D. Kasprian G. Krampl E. Ulm B. Witzani L. Prayers L.. (2006). MRI of normal fetal brain development. Eur. J. Radiol. 57, 199–216. 10.1016/j.ejrad.2005.11.020 16413984

[B24] Rados M. Judas M. Kostović I. (2006). *In vitro* MRI of brain development. Eur. J. Radiol. 57, 187–198. 10.1016/j.ejrad.2005.11.019 16439088

[B25] Rakic P. (2006). A centruy of progress in corticoneurogenesis: from silver impregnation to genetic engineering. Cereb. Cortex 16, i13–i17. 10.1093/cercor/bhk036 16766705

[B26] Saleem S. N. (2013). Fetal magnetic resonance imaging (MRI): a tool for a better understanding of normal and abnormal brain development. J. Child Neurol. 28, 890–908. 10.1177/0883073813486296 23644716

[B27] Takahashi E. Dai G. Wang R. Ohki K. Rosen G. D. Galaburda A. M.. (2010). Development of cerebral fiber pathways in cats revealed by diffusion spectrum imaging. Neuroimage 49, 1231–1240. 10.1016/j.neuroimage.2009.09.002 19747553 PMC2789885

[B28] Takahashi E. Song J. W. Folkerth R. D. Grant P. E. Schmahmann J. D. (2013). Detection of postmortem human cerebellar cortex and white matter pathways using high angular resolution diffusion tractography: a feasibility study. Neuroimage 68, 105–111. 10.1016/j.neuroimage.2012.11.042 23238434 PMC4393953

[B29] Thayyil S. Sebire N. J. Chitty L. S. Wade A. Chong W. Olsen O.. (2013). Post-mortem MRI versus conventional autopsy in fetuses and children: a prospective validation study. Lancet 382, 223–233. 10.1016/s0140-6736(13)60134-8 23683720

[B30] Ulfig N. Neudörfer F. Bohl J. (2000). Transient structures of the human fetal brain: subplate, thalamic reticular complex, ganglionic eminence. Histol. Histopathol. 15, 771–790. 10963122 10.14670/HH-15.771

[B31] Vasung L. Huang H. Jovanov-Milošević N. Pletikos M. Mori S. Kostović I. (2010). Development of axonal pathways in the human fetal fronto-limbic brain: histochemical characterization and diffusion tensor imaging. J. Anat. 217, 400–417. 10.1111/j.1469-7580.2010.01260.x 20609031 PMC2992416

[B32] Vasung L. Jovanov-Milošević N. Pletikos M. Mori S. Judaš M. Kostović I. (2011). Prominent periventricular fiber system related to ganglionic eminence and striatum in the human fetal cerebrum. Brain Struct. Funct. 215, 237–253. 10.1007/s00429-010-0279-4 20953626

[B33] Zhan J. Dinov I. D. Li J. Zhang Z. Hobel S. Shi Y.. (2013). Spatial-temporal atlas of human fetal brain development during the early second trimester. Neuroimage 82, 115–126. 10.1016/j.neuroimage.2013.05.063 23727529 PMC3876574

